# Activation of the Innate Immune System in Brain-Dead Donors Can Be Reduced by Luminal Intestinal Preservation During Organ Procurement Surgery - A Porcine Model

**DOI:** 10.3389/ti.2024.13569

**Published:** 2024-10-31

**Authors:** Marc Gjern Weiss, Anne Marye de Jong, Helene Seegert, Niels Moeslund, Hanno Maassen, Camilla Schjalm, Eline de Boer, Henri Leuvenink, Tom Eirik Mollnes, Marco Eijken, Anna Krarup Keller, Gerard Dijkstra, Bente Jespersen, Søren Erik Pischke

**Affiliations:** ^1^ Department of Nephrology, Aarhus University Hospital, Aarhus, Denmark; ^2^ Department of Clinical Medicine, Aarhus University, Aarhus, Denmark; ^3^ Department of Gastroenterology and Hepatology, University Medical Centre Groningen, University of Groningen, Groningen, Netherlands; ^4^ Department of Cardiology, Aarhus University Hospital, Aarhus, Denmark; ^5^ Department of Surgery, University Medical Centre Groningen, University of Groningen, Groningen, Netherlands; ^6^ Department of Immunology, Oslo University Hospital, Oslo, Norway; ^7^ Institute of Clinical Medicine, University of Oslo, Oslo, Norway; ^8^ Research Laboratory, Nordland Hospital, Bodø, Norway; ^9^ Department of Immunology, Aarhus University Hospital, Aarhus, Denmark; ^10^ Department of Urology, Aarhus University Hospital, Aarhus, Denmark; ^11^ Department of Anaesthesiology and Intensive Care, Oslo University Hospital, Oslo, Norway

**Keywords:** brain dead donor, luminal intestinal preservation, C3a, terminal complement complex, lipopolysaccharide binding protein, innate immune system, organ procurement and porcine model

## Abstract

Organs obtained from brain dead donors can have suboptimal outcomes. Activation of the innate immune system and translocation of intestinal bacteria could be causative. Thirty two pigs were assigned to control, brain death (BD), BD + luminal intestinal polyethylene glycol (PEG), and BD + luminal intestinal University of Wisconsin solution (UW) groups. Animals were observed for 360 min after BD before organ retrieval. 2,000 mL luminal intestinal preservation solution was instilled into the duodenum at the start of organ procurement. Repeated measurements of plasma C3a, Terminal Complement Complex (TCC), IL-8, TNF, and lipopolysaccharide binding protein were analysed by immunoassays. C3a was significantly higher in the BD groups compared to controls at 480 min after brain death. TCC was significantly higher in BD and BD + UW, but not BD + PEG, compared to controls at 480 min. TNF was significantly higher in the BD group compared to all other groups at 480 min. LPS binding protein increased following BD in all groups except BD + PEG, which at 480 min was significantly lower compared with all other groups. Brain death induced innate immune system activation was decreased by luminal preservation using PEG during organ procurement, possibly due to reduced bacterial translocation.

## Graphical Abstract

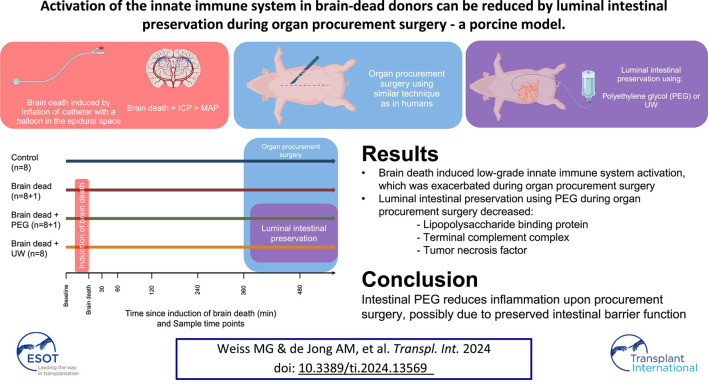

## Introduction

Deceased donors for organ transplantation comprise brain-dead donors and circulatory dead donors. Studies have established worse outcomes in donor organs obtained from brain dead donors, compared to healthy living donors [1–3] due to a multitude of factors including activation of the immune system [3–9]. The inflammatory response to brain death includes the release of damage-associated molecular patterns and activation of the innate immune system [4–6, 10]. Activation of the innate immune system in brain dead donors leads to damage to the donor organs, resulting in poorer outcomes in the recipients [3, 5, 7–9, 11, 12].

The trauma inflicted by organ procurement surgery is thought to exacerbate the initial inflammatory response in brain-dead donors, leading to additional activation of the immune system during organ procurement [13]. However, the mechanisms involved are unknown.

Although the effects of brain death on the immune system are already established at the time of organ donation decision-making, improvements can be made to limit the additional impact of organ procurement surgery.

Damage to the intestines upon brain death and possibly during organ procurement surgery leads to translocation of bacteria and bacterial remnants to the systemic circulation, which may activate and prolong activation of the innate immune system and trigger an adaptive immune response [14, 15]. Intestinal leakage can be reduced by preserving the intestinal barrier function [16], which has been demonstrated in several studies during static cold storage of intestinal grafts in rats [17–21], pigs [22] and humans [23] using luminal intestinal preservation. In these studies, luminal intestinal preservation was introduced for the preservation of intestinal grafts and was applied following vascular flush. Several clinically available organ preservation solutions have been tested for luminal intestinal preservation. University of Wisconsin static cold storage solution (UW) has been evaluated in several studies [24–26]. Polyethylene glycol (PEG) has been found to have the same preservation characteristics as UW luminal intestinal preservation [18, 19, 22].

We hypothesized that implementing a luminal intestinal intervention using established intestinal preservation fluids at the onset of organ procurement surgery in brain dead organ donors could decrease intestinal leakage and systemic immune system activation caused by the organ procurement surgery. We utilized an established porcine model of brain death to evaluate the impact of an intervention on the small intestine, consisting of luminal intestinal preservation during organ procurement surgery, using either PEG or UW solution. Our objectives were to characterize the immune response to brain death and to test two commonly used intestinal preservation solutions’ effect on this immune response.

## Materials and Methods

### Ethics and Animals

Thirtytwo female laboratory pigs (Danish landrace, Duroc, and Yorkshire crossbreed) from the same breeder, weighing 58–62 kg were planned. Two additional pigs were added later in the study due to extra pigs needed in two studies utilizing organs from this study. One pig was added to the brain dead without luminal intestinal preservation group for a project using the kidneys [27] and one pig was added to the brain dead with luminal intestinal preservation using PEG for a project using the small intestine (unpublished). All animal handling was conducted in concordance with the European Union- (directive 2010/63/EU) and local regulations. The project was approved by the Danish Animal Experiments Inspectorate (reference number: 2019-15-0201-00157).

### Study Design

**FIGURE 1:**
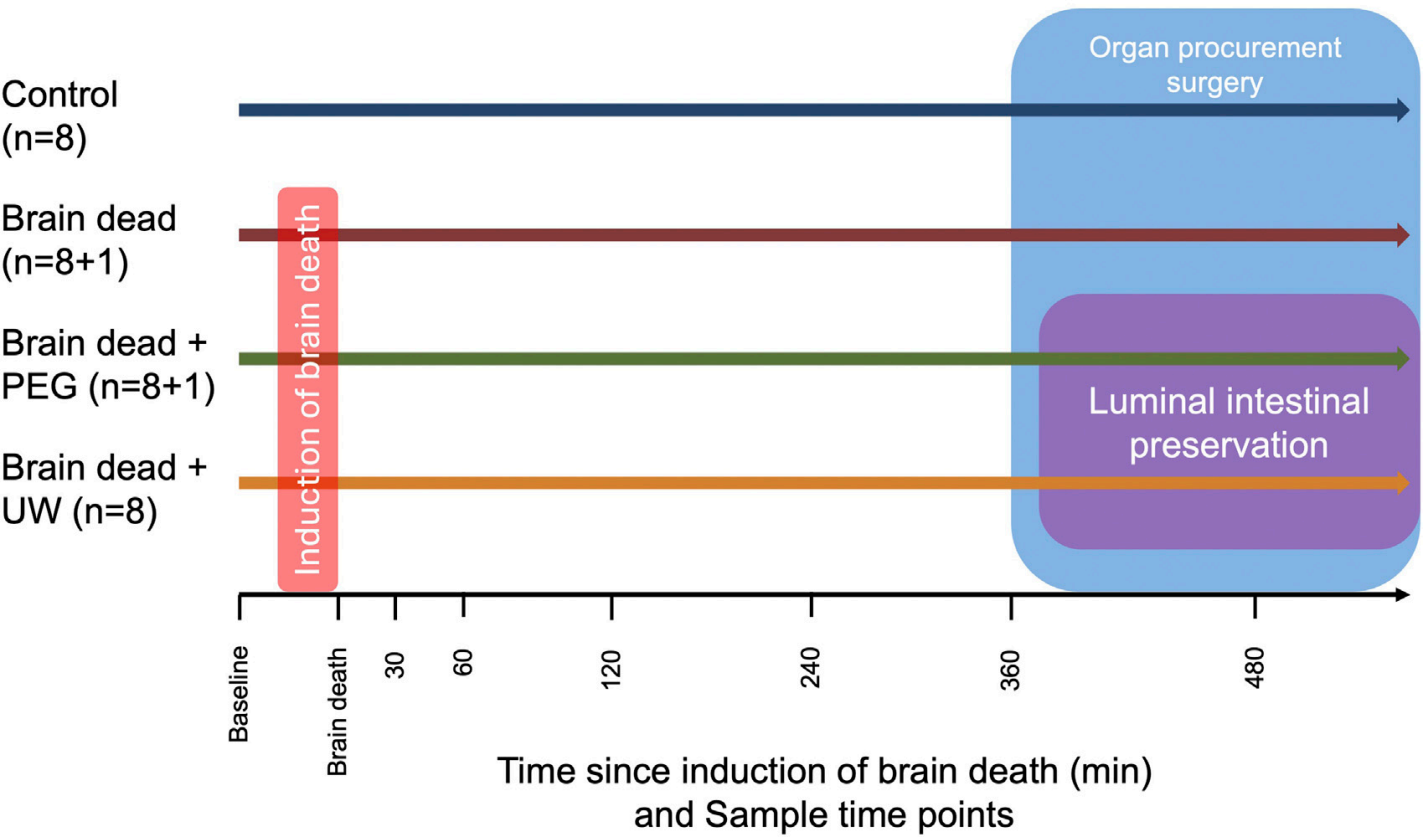
Overview of experimental groups, timeline of sample time points and interventions by groups. Animals in the brain dead, brain dead + PEG and brain dead + UW group had brain death induced while animals in the control group did not. All animals had a intracranial pressure sensor placed. In the control group, the observation period began 20 min after placing the pressure sensor to ensure approximate equal observation time. Organ procurement surgery was caried out in all groups, animals in the brain dead + PEG and brain dead + UW groups had 2,000 mL of the group appropriate solution instilled in the small intestine over approximately 2 h during surgery. Control: control group, Brain dead: brain dead without luminal intestinal intervention, Brain dead + PEG: brain dead with luminal intestinal intervention using polyethylene glycol, Brain dead + UW: brain dead with luminal intestinal intervention using University of Wisconsin solution.

Animals were divided into four groups, non-brain-dead control (Control) (n = 8), brain dead without luminal intestinal preservation group (Brain dead) (n = 8 + 1), brain dead with luminal intestinal intervention using PEG (Brain dead + PEG) (n = 8 + 1) and brain dead with luminal intestinal intervention using UW (Brain dead + UW) (n = 8) (Figure 1).

Animals were excluded from the study when one of the predefined criteria occurred at any time during the experiment: illness (diarrhea, signs of active pericarditis, peritonitis or tumors), brain death was not possible to establish, SaO_2_ < 90% with FiO_2_ of 0.3 for more than 15 min, mean arterial blood pressure < 60 mmHg for more than 15 min despite fluid resuscitation and noradrenaline infusion, death before vascular flush.

### Anesthesia

The pigs were fasted from midnight with free access to water. Pigs were sedated using “Zoletil-mix” [Zoletil 50 vet. (125 mg Tiletamin, 125 mg Zolazepam), 125 mg Xylazin, 125 mg Ketamin, 25 mg Butorphanol] intramuscularly before transport. Upon arrival at the research facility, two 18G intravenous catheters were placed, one in each ear. If necessary 50–100 mg of Propofol was administered intravenously for intubation. After intubation, anesthesia was maintained using Propofol (8 mg/kg/h) and Fentanyl (10 μg/kg/h). Ventilation was achieved with a tidal volume between 8 and 10 mL/kg and regulated to a PaCO_2_ between 5.5 and 6.5 kPa. A 14Fr urethral indwelling catheter was placed and connected to a two-chamber collection bag, allowing for precise hourly measurements of urine output.

After infusion of 1 Liter of Ringers acetate, 400 mL of blood was removed from all pigs, regardless of group allocation. This blood was used for normothermic machine perfusion of the kidneys in another study [27].

### Induction of Brain Death and Observation

After turning the pig to a prone position, an approximately 10 cm incision was made along the sagittal suture. Intracranial pressure monitoring was established in all animals using a ralk hand drill with a 5 mm drill bit, a CH5 bolt, and a NEUROVENT-PTO catheter (Raumedic, Helmbrechts, Germany). A 20 mm Hudson hand drill and back-biting forceps were used for the brain dead groups to make a burr hole. Subsequently, a CH22 Foley-type catheter (Unomedical, Lejre, Denmark) was placed in the epidural space through the burr hole.

Induction of brain death was performed according to a previously established model [28]. Briefly, the balloon of the CH22 catheter was inflated with saline, at a rate of 1 mL/min for 10 min, followed by 0.5 mL/min for an additional 10 min, and 0.25 mL/min, until a persistent negative cranial perfusion pressure (mean arterial pressure minus intracranial pressure) confirmed brain death. Following confirmation of brain death, the balloon was inflated with an additional 10 mL of saline to ensure maintenance of brain death during the entire experiment. Induction of brain death was followed by a 30-minute period with no additional interventions.

The control group only had the NEUROVENT-PTO catheter placed, the start of the observation period before organ procurement surgery began 20 min after finishing placement of the catheter, to ensure approximately equal observation time in all groups.

After these 30 min, treatment was administered for hypotension (mean arterial blood pressure < 60 mmHg: fluid bolus, noradrenaline infusion), hypertension (mean arterial blood pressure > 150 mmHg: Propofol, Labetalol), bradycardia (heart rate < 30/min: Atropine), tachycardia (heart rate > 200/min: fluid bolus, Metoprolol, Labetalol) and diabetes insipidus (urine output > 1,000 mL/h: Desmopressin) guided by an anesthesiologist trained in intensive care management and organ donor care.

### Surgical Procedure

After 360 min of observation following induction of brain death, the pig was turned back to a supine position and the organ procurement surgery commenced with a midline laparotomy. A feeding tube was inserted into the duodenum through a small incision in the ventricle’s anterior surface. The distal ileum and proximal duodenum were ligated in pigs of the brain dead + PEG and brain dead + UW groups. The small bowel was filled with 2 Liters of 4°C group-appropriate preservation fluid from the start of surgery and for the following 2 h. The filling volume was based on a presumed safe filling volume of <2 mL/cm of intestine in a 60 kg pig [22].

The abdominal organs were prepared for explantation in sequential order: kidneys, liver, and pancreas. The aorta and vena cava inferior were dissected free from the surrounding tissue and ligatures were placed around both vessels to allow for distal closure and cannulation of the abdominal aorta for vascular flush. Next, a sternotomy was performed, and the ascending aorta and the pulmonary trunk were separated and prepared for canulation.

A timeout was held, 500 IE/kg of heparin was administered and after 3 min, a St. Thomas cannula was placed in the ascending aorta. The abdominal aorta and vena cava inferior were closed distally and the abdominal aorta was canulated using a 14Fr cannula (Bridge to Life, London, England). The ascending aorta was cross-clamped, 1,000 mL of cold St. Thomas I solution was infused through the St. Thomas cannula, and the inferior vena cava was transected above the diaphragm. The abdominal aorta was cross-clamped above the liver, 4 L of cold Belzer UW Cold Storage Solution (Bridge to Life, London, England) was infused through the cannulation of the abdominal aorta, and the inferior vena cava was transected proximally to the ligature closing of the venous return from the legs. Crushed glucose ice (50 mg/mL glucose saline) was placed around all transplantable organs to provide external cooling and two suction devices were used to remove warm blood from the thorax and abdomen. Upon cardiac arrest, tissue biopsies were taken from the heart and lungs. After the vascular flush of the abdominal organs was completed, the small intestine was ligated in both ends and removed. Next, tissue samples of the liver, pancreas, and psoas muscle were obtained and the kidneys were removed.

### Blood Samples

Blood samples were collected at baseline (immediately following intubation and central venous access), time of brain death (T = 0), 30, 60, 120, 240, and 360 min after brain death. Additional blood samples were collected after 2 h of procurement surgery, prior to vascular flush (480 min after brain death). Blood was collected in EDTA vacutainer tubes and stored on ice for 30 min before centrifugation at 2.500G at 4°C for 15 min. Supernatants were aliquoted and stored at −80°C until analysis.

### Lipopolysaccharide Binding Protein, Cytokines, and Complement Factors

EDTA Plasma samples were analyzed for lipopolysaccharide binding protein (LBP) (Hycult Biotech, HK503, Uden, Netherlands), IL-8 (Merck & Co., PCYTMAG-23K, Rahway, NJ, United States), and TNF (R&D Systems, PTA00, Minneapolis, MN, United States) according to the manufacturer’s instructions. Complement components C3a and terminal complement complex (soluble TCC, sC5b-9) were assessed using highly specific in-house porcine ELISA as described previously [29]. Briefly, neoepitopes on sC5b-9 and C3a were detected by specific monoclonal capture antibodies reacting with the C3 fragment [30] and activated C9 [31], respectively. Activation levels were related to the Internation Complement Standard #2, defined to contain 1,000 complement arbitrary units (CAU) per milliliter [32]. All results are normalized to albumin to account for the differences in volume status between brain dead and non-brain dead animals.

### Tissue Samples

Snap frozen punch biopsies were taken from the heart, lungs, liver, small intestine, pancreas, kidneys, and psoas muscle at the time of removal. Frozen punch biopsies were stored at −80°C. The biopsies were homogenized and prepared for analysis for content of TNF, C3a, TCC, and IL-8 as described previously [33]. However, tissue IL-8 was not reliably detectable in >50% of samples and was thus excluded.

### Statistics

The sample size of eight animals per group was calculated based on an estimated reduction of innate immune system activation of >20% in the BD groups with luminal intestinal intervention, compared to BD without luminal intestinal intervention. Additionally, it was estimated that approximately 10% of animals would get excluded due to the above-mentioned criteria. Complete randomization was not possible due to the kidneys and intestines being used in other projects [27], requiring prior planning. Instead, subjects were randomized before each experiment between either control and brain dead or brain dead + PEG and brain dead + UW.

All analyses of blood, and tissue samples were performed blinded to group allocation.

At baseline, continuous variables were presented as mean and standard deviation when normally distributed, as assessed by quantile-quantile plots. Comparison of characteristics at baseline and of experimental characteristics was conducted using one-way analysis of variance with *post hoc* comparison of multiple means using Tukeys test. Repeated measurements of blood samples were described as median and interquartile range (IQR). Comparisons between groups and time points for repeated measurements were made with a multilevel mixed-effects linear regression model with group and time as fixed effects and individual pigs as random for all analysis, except circulating IL-8 and TNF, which were analysed using Wilcox signed-rank test, due to the non-normal distribution of values. To visualize the cytokine development from baseline to end-of-procurement, relative changes of plasma cytokine concentrations were calculated as a X-fold induction at end-of-procurement compared to baseline values. Comparison of tissue C3a, TCC, and TNF was done by Kruskal-Wallis one-way analysis of variance. Statistical significance was defined as a *p*-value of < 0.05. All statistical analyses were performed using STATA 17.0 (STATA Corp., College Station, Texas, United States) and GraphPad Prism 10.0 (GraphPad Software, Boston, Massachusetts, United States).

## Results

### Study Characteristics

A total of 30 animals remained in the study: control (n = 7), brain dead (n = 8), brain dead + PEG (n = 7), and brain dead + UW (n = 8). Four animals were excluded due to the predefined exclusion criteria [pig #7 (brain dead + PEG; peritonitis), #8 (control; death before vascular flush), #14 (brain dead + PEG; death before vascular flush) and #30 (brain dead; pericarditis and peritonitis)].

**FIGURE 2:**
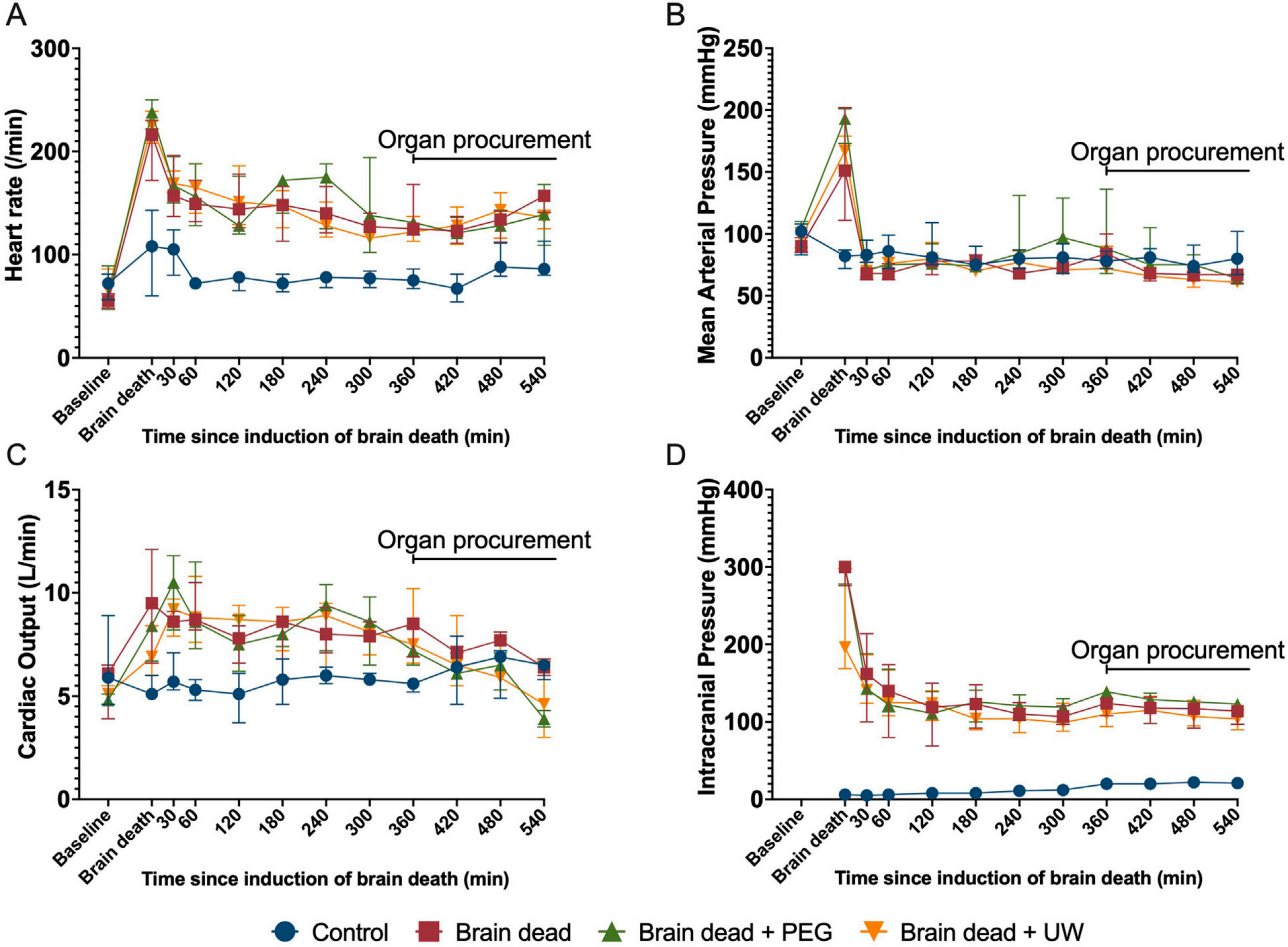
Systemic circulation during brain death, 6 h of observation, and organ procurement surgery. Heart rate **(A)**, mean arterial pressure **(B)**, continuous cardiac output **(C)**, and intracranial pressure **(D)** were kept stable in brain-dead animals after initial typical Cushing-reflex reactions upon cessation of brain perfusion due to high intracranial pressure. Animals received fluids and norepinephrine and a mean arterial pressure of above 65 mmHg was maintained throughout the experiment and organ procurement period. Control: control group, Brain dead: brain dead without luminal intestinal intervention, Brain dead + PEG: brain dead with luminal intestinal intervention using polyethylene glycol, Brain dead + UW: brain dead with luminal intestinal intervention using University of Wisconsin solution. Values presented as median ± Interquartile range.

At baseline, there were no significant differences between the four groups and/or the animals excluded (Table 1). After induction of brain death, intracranial pressure was consistently above 100 mmHg, ensuring abolished perfusion of the brain (Figure 2). Brain death resulted in a significantly increased heart rate (*p* ≤ 0.001) and cardiac output (*p* = 0.005), compared to controls. This difference persisted throughout the experiment. Mean arterial pressure was kept within predefined limits using fluid boluses and vasoactive drugs, mainly in the brain dead groups. The animals were kept stable throughout the observation period (Figure 2).

**TABLE 1:** Baseline characteristics. Characteristics of experimental animals after anesthesia and establishing of invasive monitoring.

	Control	Brain dead	Brain dead + PEG	Brain dead + UW	Excluded	*p*-value
	(n = 7)	(n = 8)	(n = 7)	(n = 8)	(n = 4)	
Animal weight, kg	63.0 (3.6)	62.5 (4.1)	66.0 (9.6)	60.2 (1.6)	61.8 (2.2)	0.32
Temperature, C°	37.4 (0.6)	37.0 (0.6)	37.0 (0.8)	36.9 (0.5)	37.4 (0.4)	0.38
Circulation						
Heart rate/min	73 (18)	60 (21)	66 (22)	71 (18)	84 (25)	0.41
Systolic blood pressure, mmHg	119 (11)	112 (11)	125 (17)	117 (16)	123 (18)	0.56
Diastolic blood pressure, mmHg	78 (14)	71 (10)	82 (16)	77 (12)	81 (15)	0.63
Mean arterial blood pressure, mmHg	97 (15)	90 (9)	100 (17)	96 (14)	98 (18)	0.76
Continuous cardiac output, L/min	6.5 (2.2)	5.5 (1.4)	4.9 (0.7)	5.0 (0.6)	4.9 (0.1)	0.45
Mixed venous O_2_, %	63 (9)	65 (3)	63 (6)	65 (5)	64 (8)	0.98
Respiration						
End tidal CO_2_, kPa	6.1 (0.6)	6.1 (0.7)	5.9 (0.8)	5.9 (0.6)	6.1 (1.0)	0.95
Saturation, f	1.00 (0.01)	0.88 (0.32)	0.99 (0.01)	0.99 (0.01)	0.99 (0.02)	0.55
Arterial blood gas						
pH	7.45 (0.03)	7.46 (0.05)	7.44 (0.05)	7.47 (0.06)	7.47 (0.05)	0.76
Lactate, mmol/L	1.3 (0.6)	1.3 (0.3)	1.4 (0.5)	1.3 (0.1)	1.0 (0.2)	0.67
Bicarbonate, mmol/L	31.8 (0.8)	31.4 (2.9)	30.2 (1.4)	31.5 (1.3)	31.8 (1.1)	0.45
Glucose, mmol/L	6.5 (1.0)	6.2 (1.0)	6.0 (0.6)	6.1 (0.8)	5.9 (1.0)	0.86
Potassium, mmol/L	3.72 (0.17)	3.63 (0.13)	3.76 (0.14)	3.73 (0.18)	3.80 (0.08)	0.41
Sodium, mmol/L	141 (2)	141 (1)	141 (1)	140 (2)	140 (1)	0.60
Biochemistry						
Hemoglobin, mmol/L	6.6 (0.4)	6.1 (0.6)	6.2 (0.3)	6.0 (0.5)	6.2 (0.4)	0.19
Leukocytes, ×10^9^/L	16.7 (1.9)	14.6 (2.6)	15.2 (4.4)	15.6 (1.6)	19.9 (3.1)	0.085
Thrombocytes, ×10^9^/L	311 (85)	272 (67)	321 (105)	284 (74)	305 (46)	0.77
Alanine transferase, mmol/L	70.0 (11.9)	71.6 (27.0)	68.7 (16.0)	77.1 (16.0)	77.3 (22.5)	0.90
Lactate dehydrogenase, U/L	574 (149)	682 (244)	545 (68)	631 (160)	699 (201)	0.68
Creatinine, µmol/L	102 (19)	106 (29)	120 (24)	114 (25)	112 (18)	0.70
Urea, mmol/L	1.87 (0.06)	1.77 (0.29)	2.00 (0.36)	2.06 (0.53)	2.05 (0.64)	0.88
Albumin, g/L	10.7 (1.1)	11.3 (1.3)	10.7 (1.3)	10.8 (1.0)	11.0 (0.0)	0.87

All values are presented as mean (standard deviation). Comparison between groups using one-way analysis of variance.

There were no significant differences regarding the duration of the experiment, brain death induction, organ retrieval, or vascular flush between the groups (Table 2). The total amount of intravenous fluids given and diuresis throughout the experiment were higher in brain dead compared with non-brain dead groups but similar between brain dead groups (Table 2). In the brain dead groups, the sodium concentration increased steadily from the time of brain death. In the brain dead + UW group, the serum potassium content increased upon UW luminal instillation from 3.8 (95% CI: 2.7; 4.9) to 5.7 (95% CI: 5.7; 5.8) mmol/L at the end of procurement surgery. Additional blood gas parameters were within acceptable ranges throughout the observation period (Supplementary Figure S1).

**TABLE 2:** Experimental characteristics. Characteristics of experiments and end of surgery.

	Control	Brain dead	Brain dead + PEG	Brain dead + UW	Control vs. Brain dead
	(n = 7)	(n = 8)	(n = 7)	(n = 8)	(*p*-value)
Total experiment duration, h:m	11:16 (00:19)	11:30 (00:31)	10:53 (00:21)	11:14 (00:27)	0.071
Duration of brain death induction, h:m	N/a	00:21 (00:11)	00:17 (00:08)	00:21 (00:06)	0.781
Duration of organ retrieval surgery, h:m	03:20 (00:16)	03:20 (00:32)	03:04 (00:20)	03:06 (00:19)	0.379
Duration of vascular flush h:m	00:10 (00:02)	00:09 (00:01)	00:10 (00:01)	00:11 (00:03)	0.452
Total amount of fluid given, l	6.7 (1.0)	9.6 (1.7)*	8.7 (1.3)	9.2 (1.6)*	0.003
Total amount of diuresis, l	0.9 (0.2)	3.8 (1.1)*	3.3 (1.4)*	3.6 (1.3)*	<0.001
Potassium, mmol/L	4.1 (0.2)^#^	3.9 (0.3)^#^	4.0 (0.3)^#^	5.5 (0.4)	<0.001
Sodium, mmol/L	136 (1.7)	148 (3.5)*	144 (3.7)*	144 (4.2)*	<0.001

All values are presented as mean (standard deviation). Comparison between groups using one-way analysis of variance and *post hoc* comparison of multiple means using Tukey test; N/a, Not available. * Signifies statistical significance of the group compared with the control group only; ^#^ Signifies statistical significance of the group compared with the brain dead + UW group only.

### Plasma Analyses of Complement Activation and Cytokines

**FIGURE 3:**
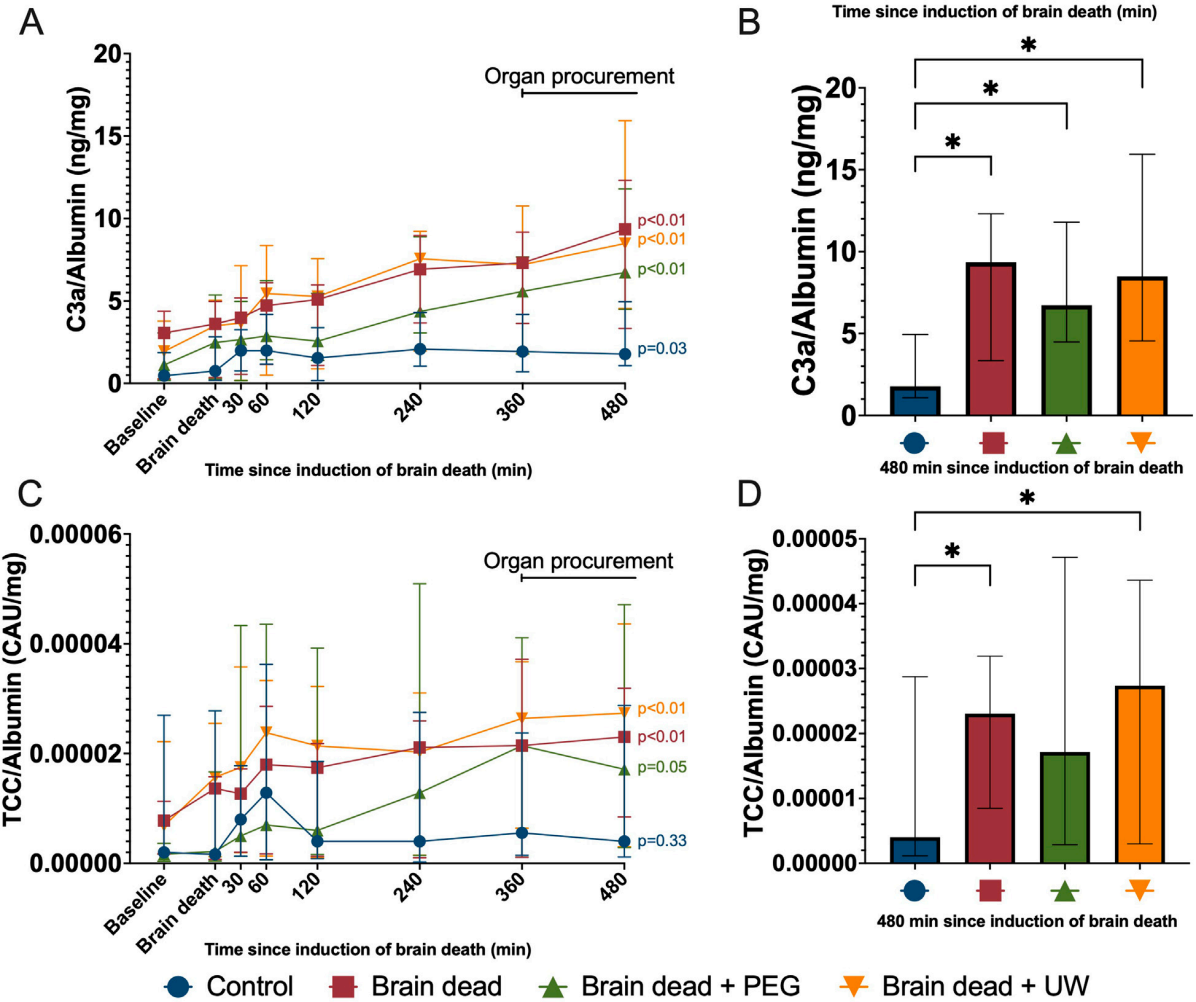
Brain death-induced complement activation irrespective of luminal intervention. Brain death led to a significant increase of plasma C3a **(A)** in all groups and TCC **(C)** in the brain dead and brain dad + UW groups compared to baseline. At the end of organ procurement, C3a was significantly higher in all brain dead groups, compared to control **(B)**. TCC **(D)** was significantly higher in brain dead and brain dead + UWgroups compared to control. Control: control group, Brain dead: brain dead without luminal intestinal intervention, Brain dead + PEG: brain dead with luminal intestinal intervention using polyethylene glycol, Brain dead + UW: brain dead with luminal intestinal intervention using University of Wisconsin solution, C3a: Complement 3a, TCC: Terminal complement complex. Values presented as median ± Interquartile range. Mixed model. *p < 0.05.

**FIGURE 4:**
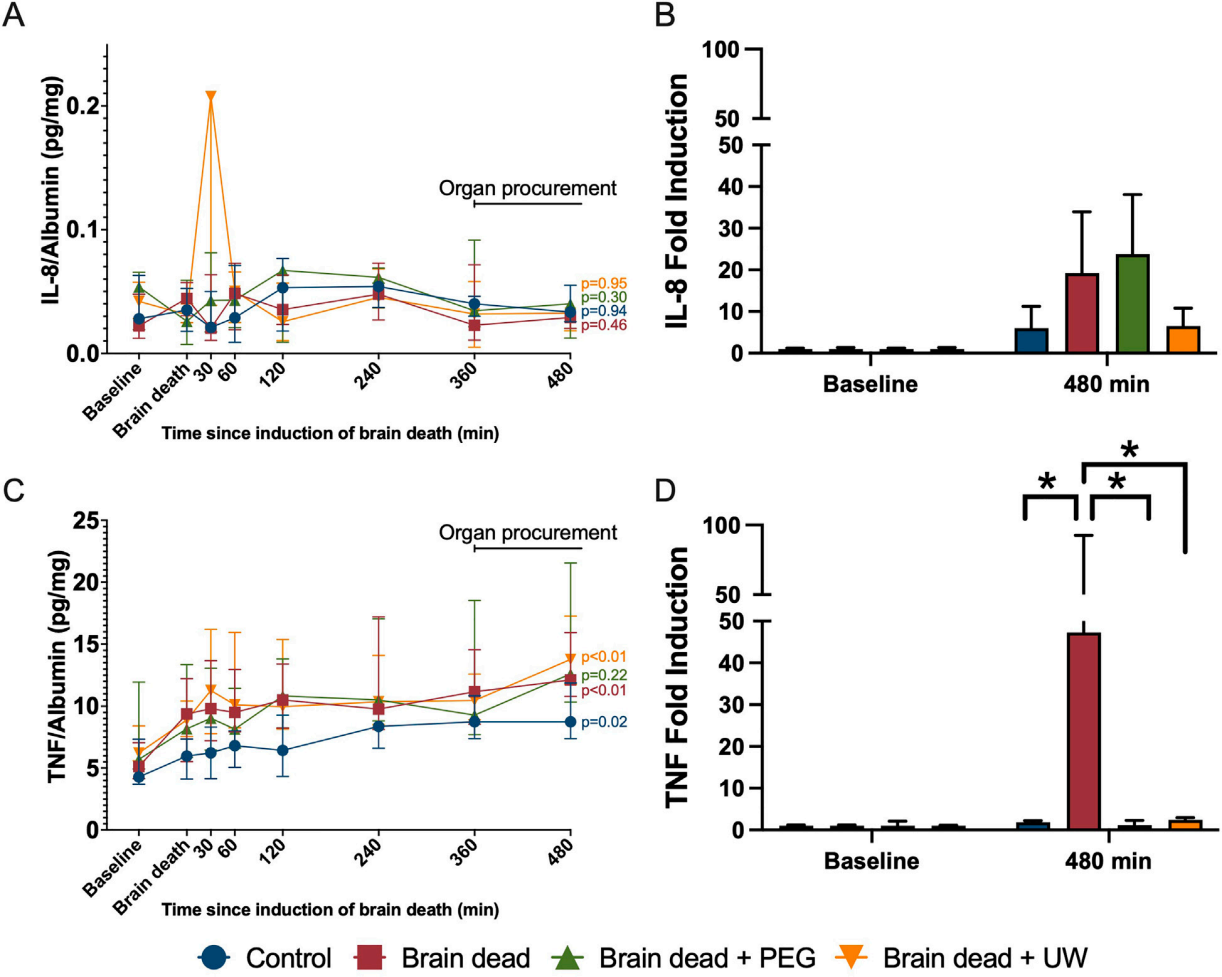
Luminal intervention prevented brain death induced release of cytokines. Brain death did not significantly increase IL-8 in any groups compared to baseline **(A)**. Brain death led to a significant increase of plasma TNF in all groups except the brain dead + PEG group com pared to baseline **(C)**. At the end of organ procurement, IL-8 **(B)** was non-significantly higher in brain-dead animals, while TNF **(D)** was significantly higher in the brain dead group compared to all other groups **(D)**. Control: control group, Brain dead: brain dead without luminal intestinal intervention, Brain dead + PEG: brain dead with luminal intestinal intervention using polyethylene glycol, Brain dead + UW: brain dead with luminal intestinal intervention using University of Wisconsin solution, IL-8: Interleukine 8, TNF: Tumor necrosis factor. Values presented as median ± Interquartile range **(A, C)** and **(B, D)** X-fold induction comparing cytokine plasma values at 480 min to baseline values as mean ± standard error of the mean. Mixed model. *p < 0.05.

Total albumin-corrected complement C3a increased following induction of brain death in all groups, compared to baseline values (Figure 3A). At 480 min, significantly higher levels of C3a were observed in the brain dead (*p* = 0.0005), brain dead + PEG (*p* = 0.0018), and brain dead + UW (*p* < 0.001) groups compared with the control group (Figure 3B). Total albumin-corrected TCC increased following induction of brain death in the brain death and brain death + UW groups compared to baseline values (Figure 3C). At 480 min, significantly higher levels of TCC were observed in the brain dead (*p* = 0.05) and brain dead + UW (*p* = 0.023) groups compared to the control group (Figure 3D). Circulating levels of IL-8 were comparable at baseline between groups and remained comparable throughout the observation period with a modest increase until the end of the experiment (Figure 4A). Circulating levels of TNF were comparable at baseline between groups. They increased following induction of brain death in all groups, significantly in the control, brain dead, and brain dead + UW groups, but not in the brain dead + PEG group (Figure 4C). Luminal intervention with UW and PEG significantly reduced TNF increase, but not IL-8 at 480 min compared with controls (Figures 4B, D).

### Tissue Samples

Tissue TCC was significantly higher in kidneys in the brain dead + PEG group compared with the brain dead + UW group. No other differences were found for tissue levels of C3a, TCC, and TNF (Supplementary Figure S2).

### Lipopolysaccharide Binding Protein

**FIGURE 5:**
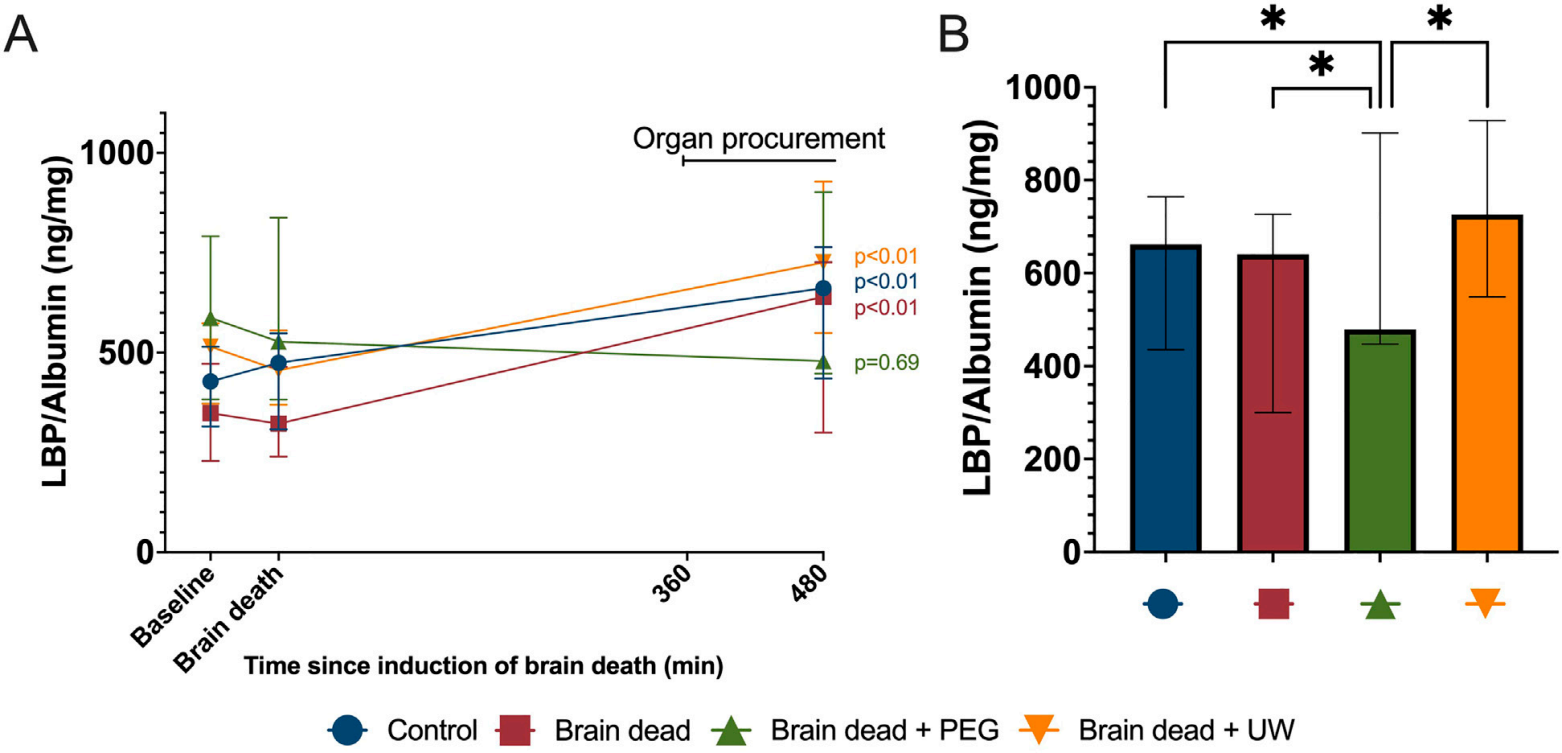
Procurement surgery-induced release of lipopolysaccharide binding protein in plasma was reduced by intestinal PEG intervention. Procurement surgery led to a significant increase in lipopolysaccharide binding protein (LBP) in all groups except the PEG intervention group **(A)**. At the end of organ procurement, lipopolysaccharide binding protein was significantly lower in the brain dead + PEG-group compared to all other groups **(B)**. Control: control group, Brain dead: brain dead without luminal intestinal intervention, Brain dead + PEG: brain dead with luminal intestinal intervention using polyethylene glycol, Brain dead + UW: brain dead with luminal intestinal intervention using University of Wisconsin solution, LBP: Lipopolysachharide binding protein. Values presented as mean ± 95% Confidence Interval. Mixed model. *p < 0.05.

No significant differences were observed at baseline or at the time of induction of brain death for LBP, (Figure 5A). Levels of LBP did not increase significantly in the brain dead + PEG group compared to the level at induction of brain death (*p* = 0.69). In contrast, all other groups increased significantly compared to baseline (*p* < 0.01) (Figure 5A). At 480 min, levels of LBP in the brain dead + PEG group were significantly lower compared to the control (*p* = 0.012), brain dead (*p* = 0.012), and brain dead + UW (*p* = 0.0002) groups (Figure 5B).

## Discussion

We showed in a porcine brain death model that intervention on the small intestine using PEG or UW fluid reduced systemic inflammation upon procurement surgery. Brain death itself induced low-grade systemic complement activation in all groups and procurement surgery led to an increase in systemic inflammation. Intestinal treatment upon procurement surgery with PEG reduced systemic LBP, TCC, and TNF. UW treatment reduced TNF but led to a significant rise in serum potassium.

Brain death affects the whole body and leads to hormonal, circulatory, and respiratory disturbances [34]. In this study, brain dead animals were comparable to non-brain dead animals concerning systemic circulation and organ function. This was achieved by medical interventions frequently used in intensive care settings and organ donor care. Brain death as well as intensive care treatment affects the immune response. We observed increasing levels of C3a, TCC, IL-8, and TNF in all animals following anesthesia and instrumentation, which is in line with previous observations [35, 36]. However, this increase was either transient or remained at low levels in the control group, confirming previous findings, that brain death, not the intensive care setting, is the main driving force of systemic immune system activation [5].

Systemic parameters of complement activation increased significantly in the brain dead groups following induction of brain death with a continuing increase of C3a and TCC throughout the observation period. These findings are in line with previous observations of the immunological response to brain death, which includes local and systemic immune responses [3–5, 7–9, 11, 12]. These responses are likely due to a combination of internal factors, such as general immune reaction, and external factors, such as the mechanism of brain death, hospital interventions, and length of stay in an intensive care unit [5, 28, 37–39]. Systemic inflammation with an increase of the pro-inflammatory protein TNF was also observed, but the observed level was low compared to e.g., systemic infection as observed in sepsis [40, 41]. However, even low-grade systemic complement activation and inflammation have been associated with long-term reduced organ function [42]. Most immunological markers increased only in the brain-dead groups after the start of the organ procurement surgery. We speculate that the initial trauma of brain death predisposes the immune system to react to the surgical trauma of organ procurement. This might be due to low-grade complement system activation.

Luminal intestinal instillation of PEG prevented the increase of TCC and TNF. PEG is part of the IGL-1 preservation solution and experimental as well as one retrospective clinical observational study have shown beneficial effects of PEG on intestinal integrity and barrier function [43, 44]. Intestinal PEG has previously been tested during storage of intestinal grafts and has shown improved preservation in animal models using rats [18–21] and humans [23]. Luminal intestinal instillation of UW only prevented the increase of TNF, while significantly increasing systemic potassium levels due to the high potassium content. High systemic potassium levels could lead to missed donations due to cardiac arrhythmias and potential cardiac arrest.

Luminal PEG significantly decreased systemic LBP, which is generated upon gram-negative and -positive bacteria stimulation [45]. TNF induction is lipopolysaccharide and LBP-dependent [46]. Although a causal link can not be made, the translocation of intestinal bacteria to the systemic circulation during procurement surgery has been hypothesized to induce inflammation and reduce donor-organ quality [47]. A previous study in rats showed that LBP increases upon procurement surgery and liver transplantation and that LBP inhibition reduces TNF levels [48]. PEG installation in the intestine might reduce this translocation by preserving intestinal barrier function [23].

However, both intestinal preservation groups showed reduced levels of TNF to the same levels as non-brain death animals, indicating reduced immune system activation in both luminal intestinal intervention groups. The effect of UW on TNF seems to be intestinal barrier independent, due to the increase in LBP, and might be explained by a rapid increase in serum potassium levels, which has been reported to inhibit inflammatory responses [49]. In addition, UW contains allopurinol, a known inhibitor of xanthine oxidase blocking the production of reactive oxygen species [50] and decreasing the production of TNF and other cytokines [51].

Both intestinal preservation groups had fluid instilled at 4°C, which could help protect the intestine from ischaemic damage during the procurement surgery due to a moderate hypothermic effect [52]. However, the intestinal temperature during surgery was not assessed and the comparable levels of LBP in the UW group compared with both control groups suggest that the intestinal barrier function was preserved by a mechanism different from hypothermia.

Luminal intestinal application of PEG is easy to implement through an already available nasogastric tube during organ procurement surgery. PEG is cheap, requires no special equipment, is clinically available as a laxative, and has no serious side effects. Additionally, the potential mechanism of protection, in the form of preserved intestinal barrier function is feasible, and negative affection of transplantable organs is highly unlikely. On the contrary, luminal intestinal application of UW did not offer the same potential benefits compared to PEG, it is expensive and presents a potential risk to the donor due to the increase in plasma potassium caused by the UW solution itself.

Intestinal leakage caused by the trauma of either circulatory- or brain death has been suggested as a possible contributory factor to worse outcomes for recipients of organs from deceased compared to healthy living donors [15, 53]. Several mechanisms have been proposed for intestinal leakage such as prolonged hypotension and hypoperfusion [54–57], mesenteric lymphatic system activation [58], and lymphocyte infiltration into the intestinal wall [15, 57]. Future clinical studies should evaluate the effect of luminal instillation of PEG at the start of organ procurement surgery on graft function and survival to improve long-term recipient survival.

This study has limitations. The withdrawal of blood early in the experiment was necessitated by use in another experiment [27]. It is possible that this exacerbated the catecholamine storm induced by brain death, which may lead to hemodynamic collapse [59]. The blood withdrawal might also have contributed to the hypovolemia induced by brain death, leading to increased use of noradrenaline and fluid to keep the animals hemodynamically stable [28, 38]. However, the blood volume removed was below 15% of total blood volume and a standard protocol of volume resuscitation and vasopressor was used. In addition, the removal of the same amount of blood in the control group did not lead to increased inflammation compared to baseline. Due to logistic reasons, randomization was only partial and the surgeon was not blinded for the intervention, which may introduce bias. However, all analyses were performed blinded to group allocation and by others than the surgeon.

Brain death induces low grade innate immune system activation, which exacerbates during organ procurement surgery. Intestinal preservation reduces systemic inflammation and PEG appears to be a better strategy compared to UW solution. PEG preservation reduces the response to intestinal bacterial translocation, which might be causative for reduced systemic inflammation during organ procurement surgery.

## Data Availability

The raw data supporting the conclusions of this article will be made available by the authors, without undue reservation.

## Supplementary Material

Supplementary Figure S1 Arterial bloodgas derived pH, lactate, sodium, and potassium. **(A)** pH remained stable in the upper normal range, with a transient decrease following the induction of brain death due to an increase in lactate. **(B)** Lactate increased upon brain death and remained elevated compared to controls. **(C)** Sodium increased slowly in the brain-dead groups following induction of brain death. Intestinal intervention showed no effect on pH, lactate, and sodium **(A–C)**. **(D)** Potassium remained within normal range in all groups except the brain-dead group receiving luminal intestinal University of Wisconsin solution, which showed a rapid increase of potassium following luminal intestinal intervention. Control: control group, Brain dead: brain dead without luminal intestinal intervention, Brain dead + PEG: brain dead with luminal intestinal intervention using polyethylene glycol, Brain dead + UW: brain dead with luminal intestinal intervention using University of Wisconsin solution, Na: Sodium, K: Potassium. Values presented as mean ± 95% Confidence Interval. Mixed model.

Supplementary Figure S2 Effect of brain death and luminal intestinal intervention of organ markers of inflammation. Tissue TCC, C3a and TNF concentrations were generally low and only mildly affected by brain death. Significant differences were observed between kidneys in the intestinal intervention groups using PEG and UW for TCC. Control: control group, Brain dead: brain dead without luminal intestinal intervention, Brain dead + PEG: brain dead with luminal intestinal intervention using polyethylene glycol, Brain dead + UW: brain dead with luminal intestinal intervention using University of Wisconsin solution; C3a, Complement 3a; TCC, Terminal complement complex; TNF, Tumor necrosis factor. All values presented as median ± interquartile range. **p* < 0.05.
