# Supplementary figures and images for: Activation of the Innate Immune System in Brain-Dead Donors Can Be Reduced by Luminal Intestinal Preservation During Organ Procurement Surgery - A Porcine Model
Source: Transpl Int. 2024 Oct 31;37:13569. doi: 10.3389/ti.2024.13569 (PMC11560447; doi:10.3389/ti.2024.13569)

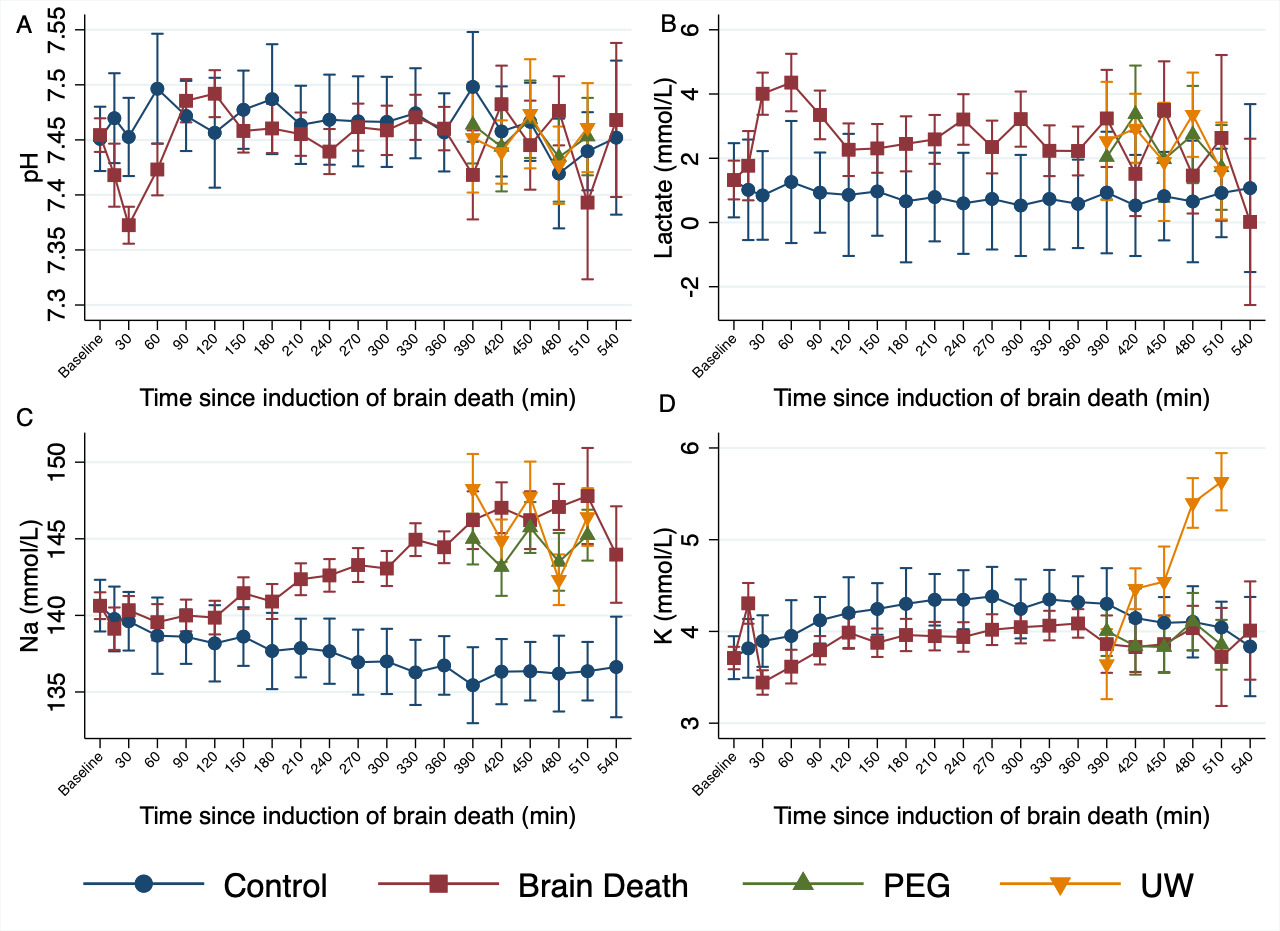

Supplement: Supplementary file 1 [file DataSheet1.zip › Supplementary figure 1.tif]

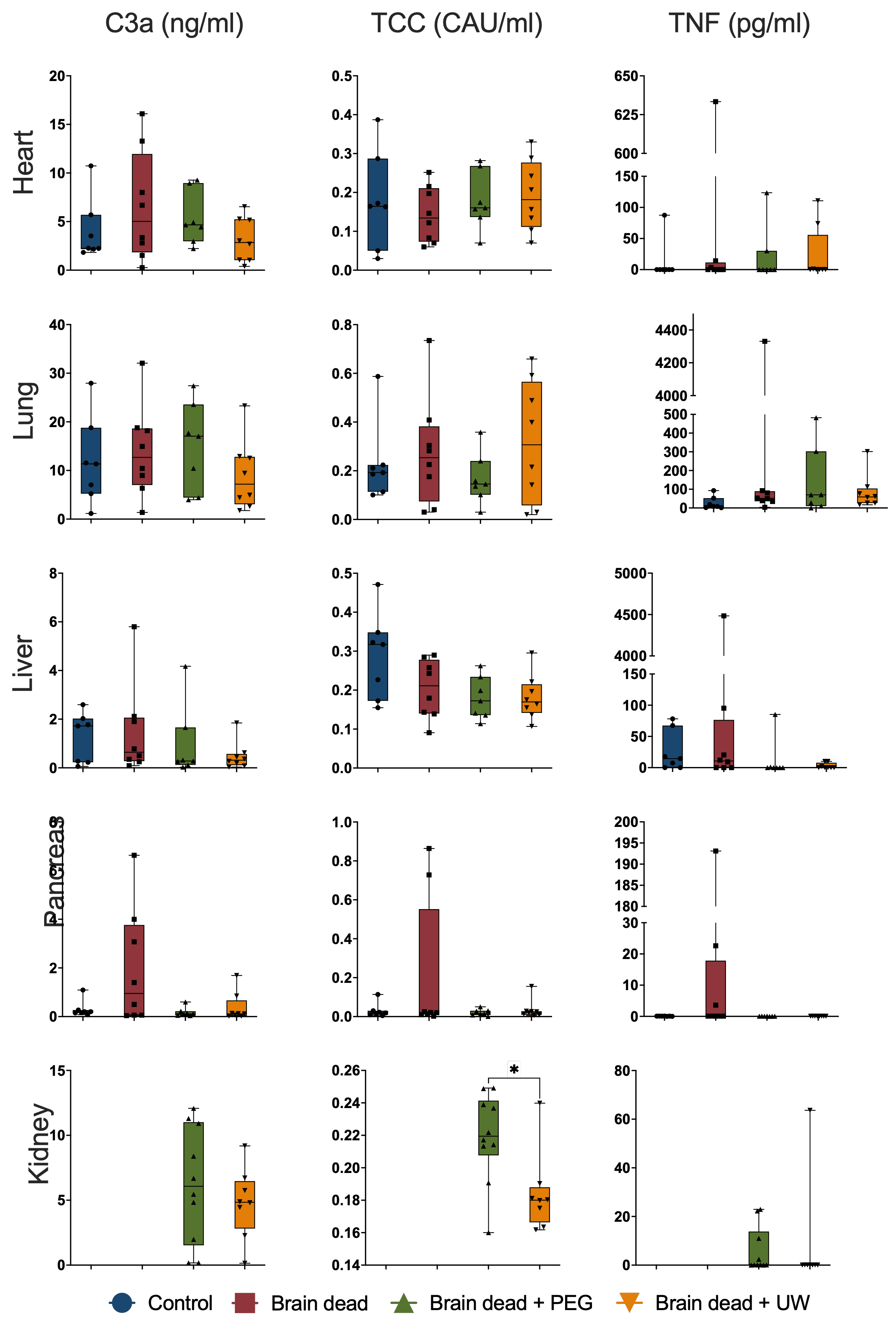

Supplement: Supplementary file 1 [file DataSheet1.zip › Supplementary figure 2.tiff]
